# Extrapolating Sentinel Surveillance Information to Estimate National COVID Hospital Admission Rates: A Bayesian Modeling Approach

**DOI:** 10.1111/irv.70026

**Published:** 2024-10-23

**Authors:** Owen Devine, Huong Pham, Betsy Gunnels, Heather E. Reese, Molly Steele, Alexia Couture, Danielle Iuliano, Darpun Sachdev, Nisha B. Alden, James Meek, Lucy Witt, Patricia A. Ryan, Libby Reeg, Ruth Lynfield, Susan L. Ropp, Grant Barney, Brenda L. Tesini, Eli Shiltz, Melissa Sutton, H. Keipp Talbot, Isabella Reyes, Fiona P. Havers

**Affiliations:** ^1^ Eagle Global Scientific LLC Atlanta Georgia USA; ^2^ CDC, National Center for Immunization and Respiratory Diseases Atlanta Georgia USA; ^3^ CDC, Global Health Center Atlanta Georgia USA; ^4^ CDC, National Center for Emerging and Zoonotic Infectious Diseases Atlanta Georgia USA; ^5^ California Department of Public Health Richmond California USA; ^6^ Colorado Department of Public Health & Environment Denver Colorado USA; ^7^ Connecticut Emerging Infections Program Yale School of Public Health New Haven Connecticut USA; ^8^ Division of Infectious Diseases Emory University School of Medicine, Georgia Emerging Infections Program Atlanta Georgia USA; ^9^ Maryland Department of Health Baltimore Maryland USA; ^10^ Michigan Department of Health and Human Services Lansing Michigan USA; ^11^ Minnesota Department of Health St. Paul Minnesota USA; ^12^ New Mexico Department of Health Santa Fe New Mexico USA; ^13^ New York State Department of Health Albany New York USA; ^14^ University of Rochester School of Medicine and Dentistry Rochester New York USA; ^15^ Ohio Department of Health Columbus Ohio USA; ^16^ Public Health Division Oregon Health Authority Portland Oregon USA; ^17^ Vanderbilt University Medical Center Nashville Tennessee USA; ^18^ Salt Lake County Health Department Salt Lake City Utah USA

**Keywords:** COVID‐19, Population‐based surviellance, hospitalization, sentinel surviellance, Bayesian modeling

## Abstract

The COVID‐19‐Associated Hospitalization Surveillance Network (COVID‐NET) was established in March 2020 to monitor trends in hospitalizations associated with SARS‐CoV‐2 infection. COVID‐NET is a geographically diverse population‐based surveillance system for laboratory‐confirmed COVID‐19‐associated hospitalizations with a combined catchment area covering approximately 10% of the US population. Data collected in COVID‐NET includes monthly counts of hospitalizations for persons with confirmed SARS‐CoV‐2 infection who reside within the defined catchment area. A Bayesian modeling approach is proposed to estimate US national COVID‐associated hospital admission rates based on information reported in the COVID‐NET system. A key component of the approach is the ability to estimate uncertainty resulting from extrapolation of hospitalization rates observed within COVID‐NET to the US population. In addition, the proposed model enables estimation of other contributors to uncertainty including temporal dependence among reported COVID‐NET admission counts, the impact of unmeasured site‐specific factors, and the frequency and accuracy of testing for SARS‐CoV‐2 infection. Based on the proposed model, an estimated 6.3 million (95% uncertainty interval (UI) 5.4–7.3 million) COVID‐19‐associated hospital admissions occurred in the United States from September 2020 through December 2023. Between April 2020 and December 2023, model‐based monthly admission rate estimates ranged from a minimum of 1 per 10,000 population (95% UI 0.7–1.2) in June of 2023 to a highest monthly level of 16 per 10,000 (95% UI 13–19) in January 2022.

## 1 Introduction

In March 2020, the Centers for Disease Control and Prevention (CDC) initiated the COVID‐19‐Associated Hospitalization Surveillance Network (COVID‐NET) to monitor population‐based rates of COVID‐19 hospital admissions [1]. COVID‐NET is an active, population‐based sentinel surveillance system that captures laboratory‐confirmed COVID‐19‐associated hospitalizations in 98 US counties across 13 states, with data verified by COVID‐NET site staff and transmitted to CDC on a weekly basis. The catchment area population covered by COVID‐NET comprises roughly 10% of the US population. If this catchment population is assumed to be reasonably representative of all US residents, then information collected among COVID‐NET sites can be used to evaluate population‐based trends in COVID‐19‐associated hospitalization risk [2, 3]. However, even if the catchment and US populations are similar, such evaluations should be made in light of the uncertainty resulting from extrapolating information observed in the COVID‐NET catchment population to the national level.

The goal of the modeling approach presented in this paper is to enable use of data collected in COVID‐NET to estimate US COVID‐19‐associated hospitalization rates. A key component of the approach is estimation of the uncertainty in the modeled rates due to extrapolation of information observed in the catchment population to the national level. The estimates are produced using a two‐step modeling process. In the first step, a Bayesian hierarchical model is used to estimate temporal trends in observed COVID‐NET catchment population hospital admission rates. In the second step, estimates of COVID‐19‐associated hospitalization rates for the US population are produced based on the estimated model parameters. Using an assumption that the variation in hospitalization rates across COVID‐NET sites provides an estimator for the variation in US state‐level rates, the proposed approach enables estimation of the uncertainty in the derived national rate estimates resulting from catchment to national population extrapolation. In addition, the model is structured to address other sources of uncertainty including: temporal dependence among the sequentially reported COVID‐NET hospitalization counts, variation in rates across COVID‐NET sites due to unmeasured factors that could impact admissions, and uncertainty on the frequency of diagnostic testing for SARS‐CoV‐2 infection and the performance of the utilized tests.

National COVID‐19 hospitalization estimates based on the proposed model were compared with estimates from another data source (the National Hospitalization Surveillance Network [NHSN]) to evaluate the assumption of representativeness for the COVID‐NET catchment population. In addition, model estimates were also compared with those produced based on an existing model used to estimate influenza hospitalization rates in a related network (FluSurv‐NET).

## 2 Methods

### 2.1 COVID‐NET Data

COVID‐NET, along with RSV‐NET and FluSurv‐NET, is one of three platforms that comprise the Respiratory Hospitalization Surveillance Network (RESP‐NET). These systems use similar case identification methods and catchment areas to conduct surveillance for COVID‐19, respiratory syncytial virus (RSV) and influenza hospitalizations respectively. From March 2020 through May 2022, catchment areas for participating COVID‐NET hospitals covered 99 counties distributed across 14 states (California, Colorado, Connecticut, Georgia, Iowa, Maryland, Michigan, Minnesota, New Mexico, New York, Ohio, Oregon, Tennessee, and Utah). Due to discontinued reporting by one site, the COVID‐NET catchment area was comprised of 98 counties in 13 states from June 2022 onward. Hospitalized patients residing in a surveillance catchment area with a positive molecular assay or rapid antigen test for SARS‐CoV‐2 during hospitalization or within 14 days prior to admission are included as COVID‐NET cases [1]. Although the COVID‐NET system collects a wide range of information on laboratory‐confirmed COVID hospitalizations, the data utilized in these analyses were monthly counts of eligible site‐specific COVID‐19‐associated admissions reported from April 2020 through December 2023 within each of six age groups (<18, 18–49, 50–64, 65–74, 75–84, and ≥85 years) as well as the corresponding COVID‐NET site and age class–specific catchment population size.

### 2.2 COVID‐19‐Associated Hospitalizations Reported in the National Health Safety Network

The National Healthcare Safety Network (NHSN) was CDC's primary source for national‐level surveillance on COVID‐19‐associated hospitalizations in the United States during the study period [4]. In addition, NHSN data was the only surveillance activity focused on enumerating COVID‐19‐associated hospitalizations at a national level. Although there are likely inherent unquantifiable uncertainties in the NHSN reported hospitalization rates and COVID‐NET and NHSN case definitions differed, comparison of the NHSN reported rates to those produced using the proposed COVID‐NET‐based model provides a potential measure of the validity of the model estimates. In addition, such a comparison enables empirical evaluation of the assumption that hospitalization rates observed in the COVID‐NET system can be used to evaluate national trends in COVID‐associated hospitalization risk.

Evaluation of available NHSN data indicated that the median proportion of hospitals per state reporting COVID‐19‐associated admissions exceeded 90% in September 2020 and remained at that level, or higher, through December 2023 [5]. As a result, NHSN national hospitalization rates reported from September 2020 through December 2023 were used in the comparison to the model‐based estimates.

### 2.3 Estimating the Probability of Detection for Eligible COVID‐NET Admissions

The first step in the modeling process was to adjust the reported COVID‐NET admission counts to account for the possibility that a COVID‐19‐associated admission was not identified because of the lack of universal SARS‐CoV‐2 testing and the use of tests with less than 100% sensitivity [2]. Data to inform this adjustment were collected in a separate sampling activity for a subset of COVID‐NET hospitals between March 2020 and September 2021 [6]. The age class–specific testing practice data included the proportion of a random sample of inpatients with a recorded pneumonia, influenza, or COVID‐19‐like condition (PIC) who were tested for SARS‐CoV‐2 and the type of test, molecular assay or rapid antigen, that was used.

For a sequential collection of time periods, in this case months, designated by m where m = 1, …, M, let ${\mathrm{pt}}_{\mathrm{msa}}$ represent the unknown true probability of testing for SARS‐CoV‐2 among those admitted with a recorded PIC for month m, COVID‐NET site s, and age class a. Estimates for ${\mathrm{pt}}_{\mathrm{msa}}$ were defined as the observed proportion of sampled PIC inpatients, within each month, site, and age class who were tested for SARS‐CoV‐2 either during hospital stay or within 14 days prior to admission. In addition, a weighted average estimator for the sensitivity of the test used was defined for each month, site, and age class strata as

$${\mathrm{Se}}_{\mathrm{msa}}=\;{\text{wMOl}}_{\mathrm{msa}}*\mathrm{Se}\left(\mathrm{MOL}\right)+\left(\;1-{\text{wMOL}}_{\mathrm{msa}}\right)*\mathrm{Se}\left(\mathrm{RAT}\right)$$

where ${\text{wMOL}}_{\mathrm{msa}}$ is the observed proportion of all tests for SARS‐CoV‐2 infection among those with a PIC that were molecular assay for month m, site s, and age class a, $1-{\text{wMOL}}_{\mathrm{msa}}$ is the corresponding proportion of rapid antigen tests, and $\mathrm{Se}\left(\mathrm{MOL}\right)$ and $\mathrm{Se}\left(\mathrm{RAT}\right)$ are the assumed sensitivities of rapid molecular and rapid antigen tests, respectively [2]. Based on literature review (see Appendix), the sensitivity of a molecular assay test was assumed to range from 0.85 to 0.98, whereas the assumed sensitivity of a rapid antigen test ranged from 0.65 to 0.75.

Under the assumption that the probability of testing and test sensitivity are independent, an estimator for the probability of detecting a COVID‐NET eligible admission for each month, site, and age class, which will be referred to as ${\mathrm{pd}}_{\mathrm{msa}}$, was defined as

(1) $${\mathrm{pd}}_{\mathrm{msa}}=\;{\mathrm{pt}}_{\mathrm{msa}}*\;{\mathrm{Se}}_{\mathrm{msa}}$$

Uncertainty associated with the observed values for ${\mathrm{pt}}_{\mathrm{msa}}$ and ${\text{wMOL}}_{\mathrm{msa}}$ was modeled by assuming Beta prior distributions for these quantities. Prior parameter values for the assumed Beta distributions were derived based on the observed value and sample variance of the proportions [7]. Additional detail on the approach used to produce estimates for ${\mathrm{pd}}_{\mathrm{msa}}$, and the assumed uncertainty regarding that probability, is provided in Appendix.

### 2.4 Modeling Temporal Trends in Observed COVID‐NET Admission Rates

The proposed model for the time series of admission counts observed in the COVID‐NET data is based on both a conditional autoregressive (CAR) assumption to address dependency in observed hospitalizations because of the temporal order in which they were observed and site‐level random effects (REs) to account for unmeasured site‐specific factors that could lead to within‐site correlation in reported hospitalization counts. Given these assumptions, the modeling approach will be referred to as the CAR‐RE model.

Let $c_{\mathrm{msa}}^{\mathrm{Obs}}$ be the observed number of hospitalizations with a confirmed positive test for SARS‐CoV‐2 reported in the COVID‐NET system for month m, COVID‐NET site s, and age class a and $c_{\mathrm{msa}}$ be the corresponding admission count if all patients with a PIC code were tested using a test with perfect sensitivity. In the first level of the modeling process, $c_{\mathrm{msa}}^{\mathrm{Obs}}$ is assumed to be a sample from a binomial distribution with number of trials given by $c_{\mathrm{msa}}$ and probability of success, that is, detection of the COVID‐related admission, given by ${\mathrm{pd}}_{\mathrm{msa}}$ as defined in Equation (1). Note that $c_{\mathrm{msa}}$ is not observed in the COVID‐NET data and is treated as missing information to be estimated as part of the Bayesian modeling process.

In the next model level, $c_{\mathrm{msa}}$ is assumed to be a sample drawn from the Poisson distribution

$$c_{\mathrm{msa}}\;\sim \;\text{Pois}\left(\mu_{\mathrm{msa}},{\mathrm{pop}}_{\mathrm{msa}}\right)$$

where $\mu_{\mathrm{msa}}$ is the true admission rate and ${\mathrm{pop}}_{\mathrm{msa}}$ is the appropriate COVID‐NET catchment area population size. The natural log of $\mu_{\mathrm{msa}}$ is assumed to be a sample from the normal distribution

(2) $$\log\left(\mu_{\mathrm{msa}}\right)\;\sim \;N\left({\mathrm{l\mu}}_{\mathrm{ma}}+\varepsilon_{s},\sigma_{\mathrm{ma}}^{2}\right)$$

where ${\mathrm{l\mu}}_{\mathrm{ma}}$ is the average age class–specific log rate of admissions across COVID‐NET sites for month m, $\varepsilon_{s}$ is a site‐specific RE, and $\sigma_{\mathrm{ma}}^{2}$ is a month and age class–specific model level variance. The site‐specific REs are modeled as samples from a normal distribution with mean zero and variance $\delta^{2}$ and are included in Equation (2) to reflect the potential influence of unmeasured site‐level factors that could lead to within‐site clustering of the observed admission rates.

Temporal dependence among the monthly age‐specific admission rates is introduced by assuming that, within each age class, the collection of M monthly average log rates, that is the collection (${\mathrm{l\mu}}_{1a},\;{\mathrm{l\mu}}_{2a},\ldots ,\;{\mathrm{l\mu}}_{\mathrm{Ma}})$, is a sample from a multivariate normal distribution with mean ${\mathrm{l\mu}}_{a}$, the average log rate across all months within that age class, and covariance matrix $\Sigma_{a}$. The form for $\Sigma_{a}$ is assumed to reflect a one‐dimensional proper autoregressive (CAR) model [8, 9]. A similar one‐dimensional CAR structure is assumed for the age class–specific average log rates in the next model level to address possible correlation among the average log rates across age classes.

A Markov chain Monte Carlo sampling approach was used to derive estimates of the posterior model parameters. One chain was run for 45,000 iterations with a burn in of 5000 samples. The remaining 40,000 iterations were thinned to retain every second sample resulting in 20,000 samples from the estimated posterior distributions for all model parameters.

Further details on the model structure, prior assumptions, and rationale for these assumptions based on exploratory analyses of the observed COVID‐NET admission data are provided in Appendix.

### 2.5 Extrapolation of Modeling Results to Estimate National COVID Hospital Admissions

Use of the posterior parameter estimates developed using the CAR‐RE model to estimate national admission rates relied on two key assumptions. First is that posterior estimates of the monthly age class–specific log rates developed using the COVID‐NET data, ${\mathrm{l\mu}}_{\mathrm{ma}}$ in Equation (2), provide unbiased estimates of the corresponding month and age class–specific national log admission rates. The second primary assumption, which provides the basis for estimation of extrapolation uncertainty, is that the distribution of state‐level rates about the estimated national average can be estimated by the distribution of COVID‐NET site‐level rates about the corresponding COVID‐NET average.

Let the superscript $\left(i\right)$ designate the sample number for each of the posterior parameter distributions produced in the CAR‐RE modeling process where $\left(i\right)=1,\ldots ,20,000$. Now consider the ${\left(i\right)}^{\mathrm{th}}$ posterior sample for the vector of monthly log rates within a given age class, that is, the vector

$$\left({\mathrm{l\mu}}_{1a}^{\left(i\right)},\;{\mathrm{l\mu}}_{2a}^{\left(i\right)},\ldots .,{\mathrm{l\mu}}_{\mathrm{Ma}}^{\left(i\right)}\;\right)\;.$$

Note that the elements of this vector will be correlated based on the assumed CAR model reflecting temporal dependence among the observed COVID‐NET rates. Let the ${\left(i\right)}^{\mathrm{th}}$ posterior estimate for the variance of the COVID‐NET site‐level REs be given by $\delta^{2\left(i\right)}$. The log admission rate for state S, month m, and age class a is then estimated as a sample from a normal distribution such that

(3) $${\mathrm{l\mu}}_{mSa}^{\left(i\right)}\;\sim \;N\left(\;{\mathrm{l\mu}}_{\mathrm{ma}}^{\left(i\right)},\;\delta^{2\left(i\right)}+\sigma_{\mathrm{ma}}^{2\left(i\right)}\;\right)$$

where $\sigma_{\mathrm{ma}}^{2\left(i\right)}$ is the ${\left(i\right)}^{\mathrm{th}}$ sample from the estimated posterior distribution for the model‐level variance. The sampled state and age class–level monthly log rates, ${\mathrm{l\mu}}_{mSa}^{\left(i\right)}$, are designated with the large and bolded subscript S to differentiate from the log rates within month and age class estimated in the modeling step for the actual COVID‐NET sites. The variance of the assumed normal distribution given in Equation (3) is the sum of the ${\left(i\right)}^{\mathrm{th}}$ realization for the estimated variance of the REs associated with the COVID‐NET sites and the corresponding posterior estimate for the model‐level variance. This reflects the assumption that the distribution of state‐level admission rates about the monthly national average can be estimated using the distribution of site‐level rates about the average monthly COVID‐NET log rate.

The extrapolation process is completed by generating realizations for the month, state, and age class–specific COVID‐admission counts, $c_{mSa}^{\left(i\right)}$ using an assumed Poisson distribution such that

(4) $$c_{mSa}^{\left(i\right)}\sim \text{Pois}\left(\exp\left({\mathrm{l\mu}}_{mSa}^{\left(i\right)}\right),\;{\mathrm{Pop}}_{mSa}\right)$$

where ${\mathrm{Pop}}_{mSa}$ is the appropriate age class–specific population size for state S [10]. The generated values for $c_{mSa}^{\left(i\right)}$ in Equation (4) were summed across states to derive estimated national overall and age class–specific COVID admission counts. Estimates for COVID admission rates were produced by dividing these summed values by the appropriate age class population counts. National admission count and rate estimates are summarized using the median of 20,000 posterior predictive samples with a 95% uncertainty interval (UI) estimated using the highest posterior probability approach [11]. Additional detail on the methods and assumptions used to extrapolate the CAR‐RE modeling results to the national level is provided in Appendix

### 2.6 Comparison of the CAR‐RE Model to Hospitalization Rate Estimates Produced Using the FluSurv‐NET Model

The CAR‐RE modeling approach can be viewed as an extension of existing methods used to estimate national influenza hospitalization rates using data from the Influenza Hospitalization Surveillance Network (FluSurv‐NET) [2]. The FluSurv‐NET system uses similar methods for case identification and catchment areas as COVID‐NET. As in the approach presented here, the first step in developing the influenza national hospitalization estimates is to adjust the reported admission counts to reflect testing frequency and test sensitivity. However, the estimation of the uncertainty due to extrapolating the adjusted observed influenza rates from the catchment population to the national level is not addressed in the FluSurv‐NET model. That is, after adjustment for detection probability, the average monthly age class–specific rates observed in the FluSurv‐NET sites are assumed to exactly equal the corresponding national age‐specific rates. This assumption translates into assumed exact equality, within each age class, between the adjusted rate observed in the catchment population and the corresponding age‐specific rate in every state.

To facilitate comparison of the two modeling approaches, alternative COVID‐19‐associated national hospitalization rates were estimated using COVID‐NET data but ignoring the potential for extrapolation uncertainty. The first step in producing these alternative COVID‐19‐associated hospitalization rates was to adjust the observed COVID‐NET hospitalization counts to account for testing frequency and sensitivity. This adjustment was carried out by dividing the observed monthly site and age class–specific COVID‐NET hospitalization counts by the probability of detection given in Equation (1). This process was repeated 20,000 times using 20,000 samples for ${\mathrm{pd}}_{\mathrm{msa}}$ drawn from the assumed uncertainty distributions for this parameter. The resulting adjusted age class–specific hospitalization counts were then summed across COVID‐NET sites, along with the appropriate catchment populations, to produce age‐group–specific estimates of COVID‐19‐associated hospitalization rates. These COVID‐NET‐level rate estimates were then assumed to equal the corresponding national age‐group–specific hospitalization rate. Note that in this reduced approach, there was no modeling of the uncertainty due to extrapolation from the catchment to US populations. For clarity, estimates of COVID‐19‐associated hospitalization rates produced using this reduced method will be referred to as adjusted for detection uncertainty only.

## 3 Results

**FIGURE 1:**
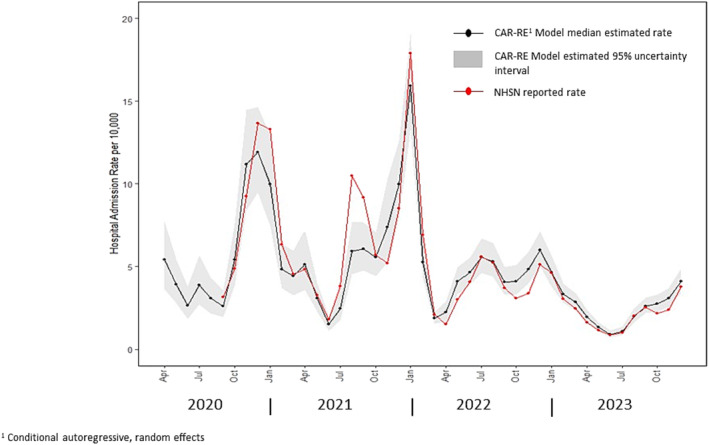
Estimated US COVID‐19‐associated hospital admission rates by month from April 2020 through December 2023 and monthly hospital admission rates reported in the National Hospital Safety Network (NHSN).

From April 2020 through December 2023, data were collected on 499,139 laboratory‐confirmed hospitalizations in the COVID‐NET catchment area. Figure 1 shows monthly estimates of the US national COVID‐hospitalization rate per 10,000 population based on these data produced using the CAR‐RE model. The solid black line in the figure indicates the median value of 20,000 posterior predictive estimates for the COVID‐19 hospital admission rate for each month, whereas the shaded band reflects the 95% uncertainty interval for the rate. Across this time interval, estimated COVID‐19 hospitalization rates ranged from a low median value of 1 admission per 10,000 population (95% UI 0.7–1.2) in June of 2023 to a highest monthly level of 16 admissions per 10,000 (95% UI 13–19) in January 2022. For comparison, hospital admission rates reported in the NHSN from September 2020 through December 2023, are also presented in Figure 1 [5]. Although for some periods, for example, August and September 2021, the NHSN reported rates lie outside the 95% UI produced using the CAR‐RE model, there is general agreement, both in time trend and magnitude, between the CAR‐RE‐based estimates and those reported in NHSN.

**FIGURE 2:**
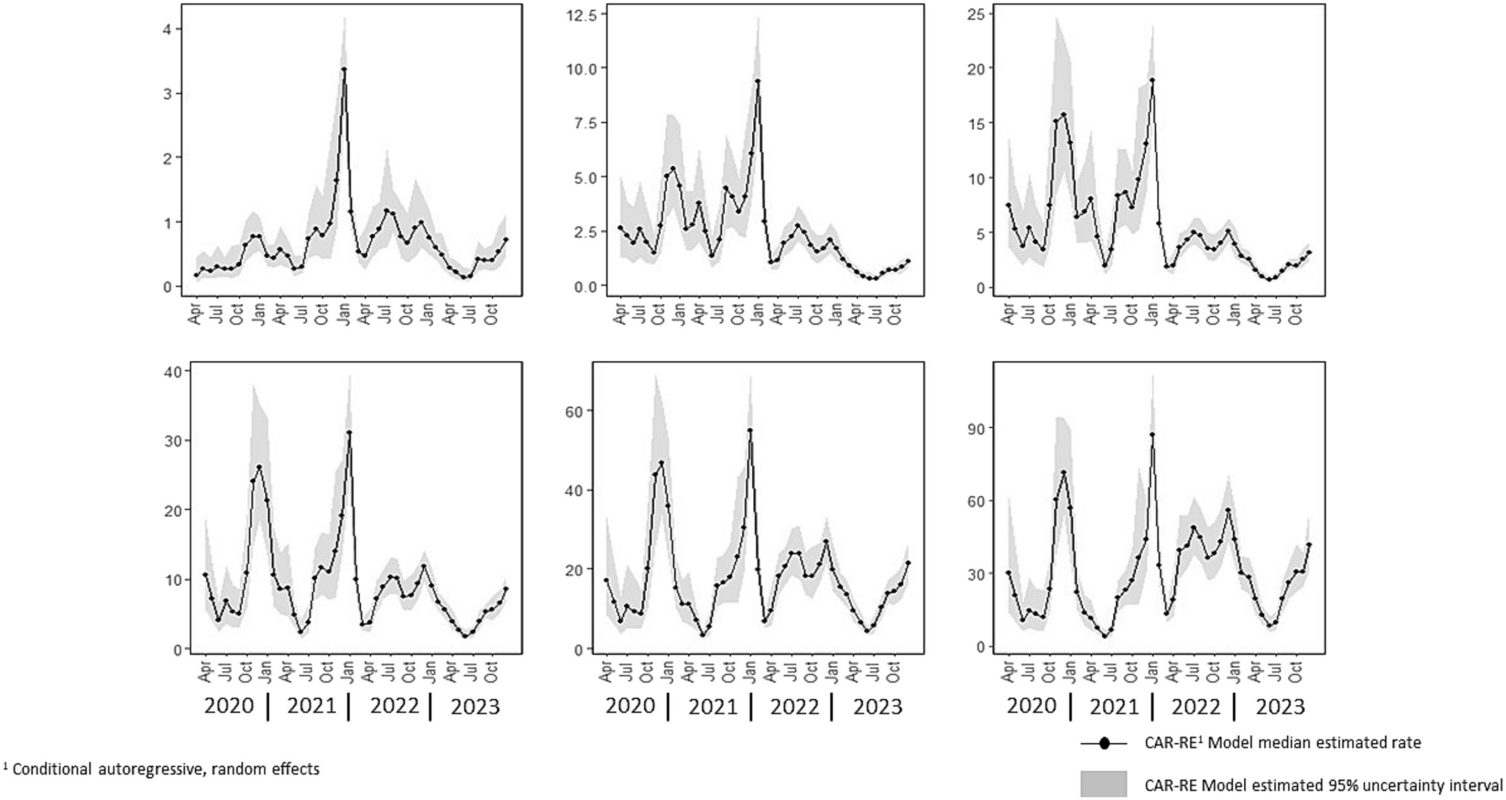
Estimated US COVID‐19‐associated hospital admission rates by month and age class from April 2020 through December 2023.

Monthly estimated COVID admission rates within six age classes are presented in Figure 2. Temporal patterns in the age class–specific estimates are generally similar when compared with the overall rate estimates apart from less pronounced peaks in late 2020 and in late 2021‐early 2022 among those in the youngest age group.

**FIGURE 3:**
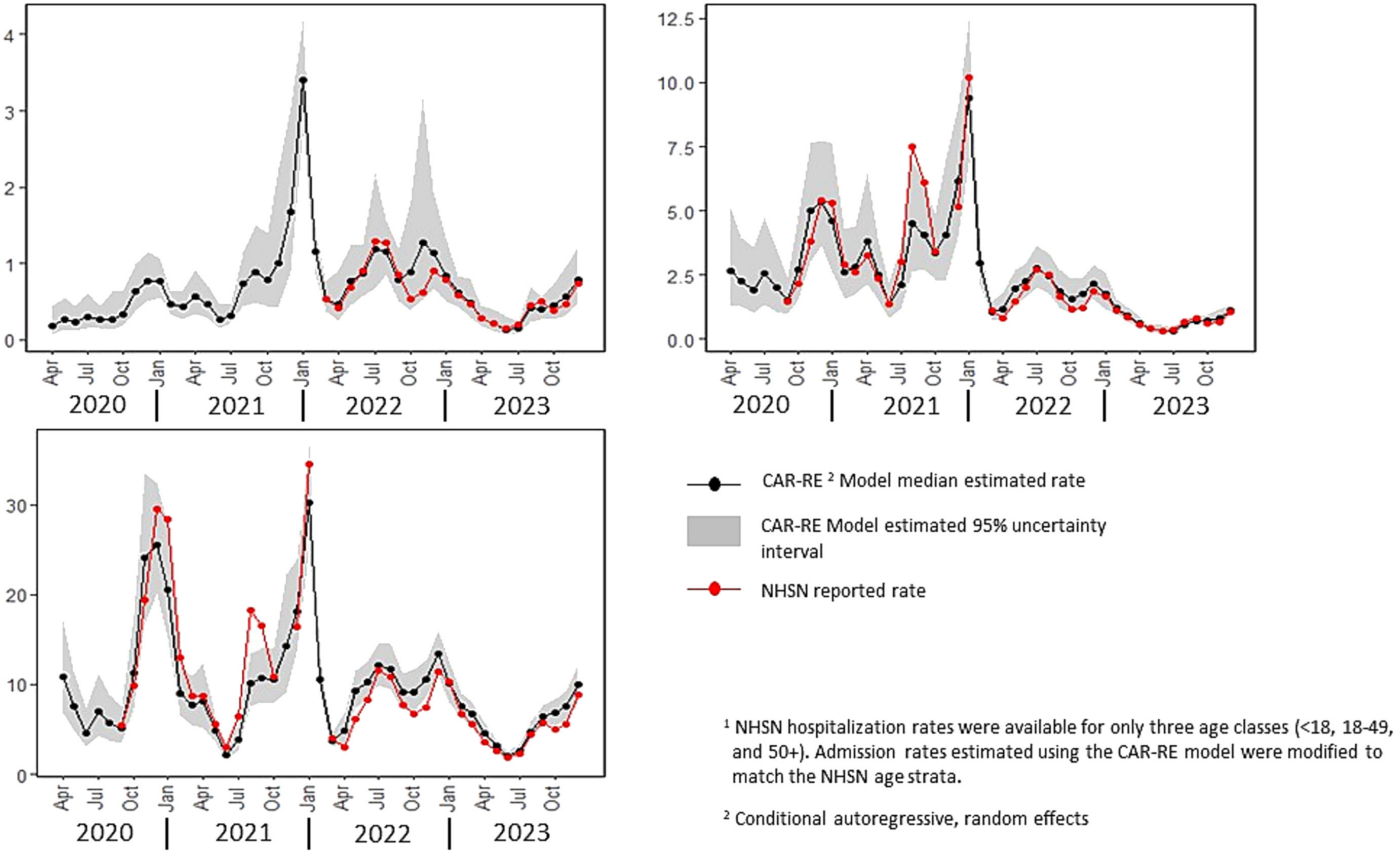
Estimated COVID‐19‐associated hospital admission rates by month and age class1 from April 2020 through December 2023 and monthly age class specific hospital admission rates reported in the National Hospital Safety Network (NHSN).

The NHSN COVID‐19‐associated hospitalization data are available in three age strata (<18, 18–49, and 50+ years), as compared with the six age classes for which rates were estimated using the CAR‐RE model [5]. To facilitate comparison of the CAR‐RE estimated and NSHN reported age‐specific rates, age class–specific hospitalization counts produced using the CAR‐RE model were combined as appropriate to match the NHSN age grouping. A comparison of the CAR‐RE and NHSN age‐specific rates is provided in Figure 3 for months in which NHSN age‐specific data were available. As with the overall national results, comparison of the age‐specific CAR‐RE estimates to rates reported in NHSN indicates general agreement in both magnitude and temporal trend.

**FIGURE 4:**
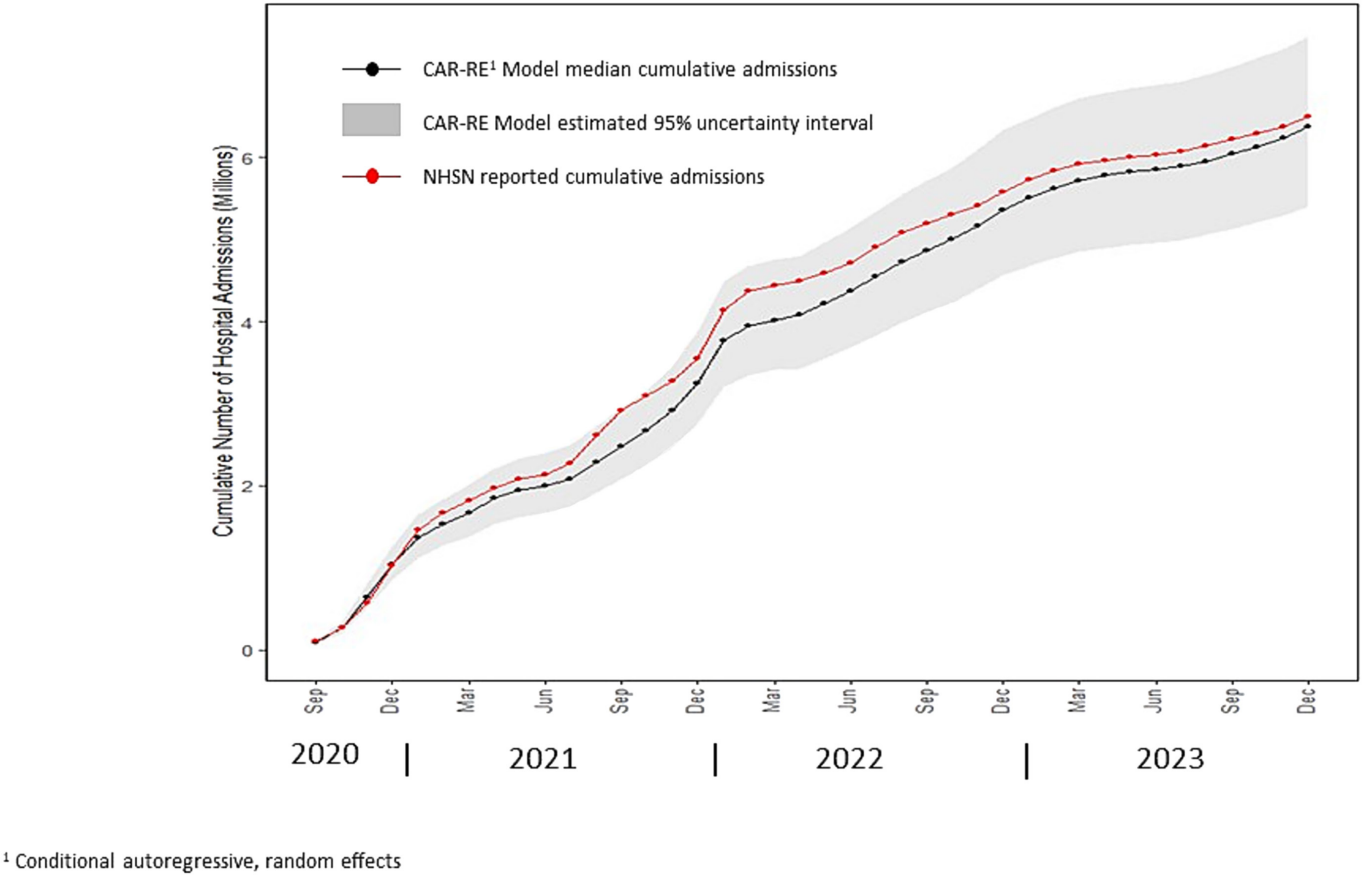
Estimated COVID‐19‐associated hospital admission rates by month from September 2020 through December 2023 and cumulative hospital admissions reported in the National Hospital Safety Network (NHSN).

The estimated cumulative number of COVID‐19‐associated hospitalization by month produced using the CAR‐RE model from September 2020 through December 2023 is shown in Figure 4. Based on the CAR‐RE modeling, an estimated 6.3 million (95% UI 5.4–7.3 million) COVID‐19‐associated hospital admissions occurred in the United States during this time interval. During the same time frame, a cumulative count of 6.3 million hospital admissions was also reported in NHSN.

**FIGURE 5:**
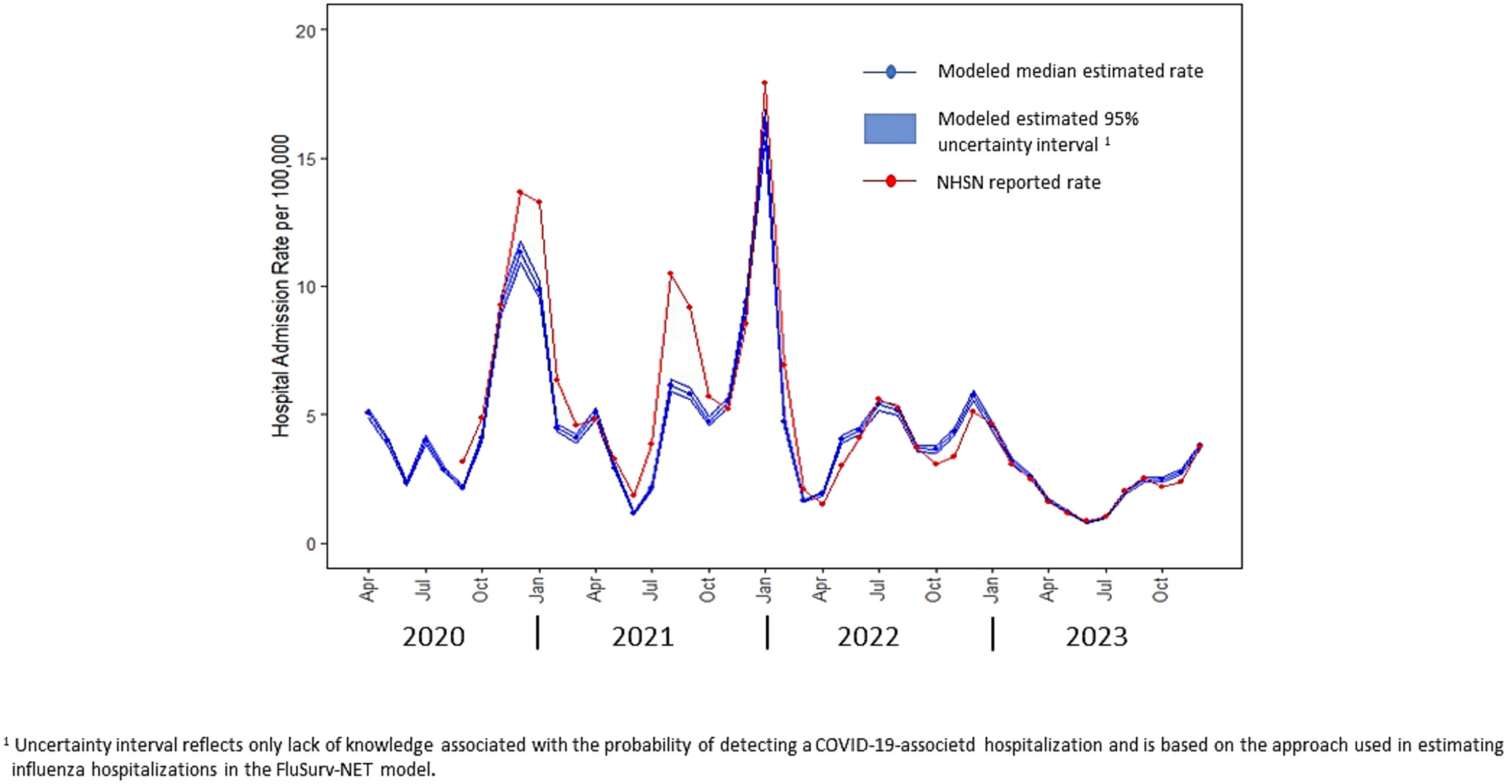
Estimated COVID‐19‐associated hospital admission rates by month from April 2020 through December 2023 after adjustment for detection uncertainly only and monthly admission rates reported in the National Hospital Safety Network (NHSN).

The impact of excluding estimation of the uncertainty resulting from extrapolation from the catchment to national populations is illustrated in Figure 5. The figure shows the detection uncertainty only estimated COVID‐19 hospital admission rates over the same time interval as that in Figure 1 but with estimation procedure altered to match that used for influenza‐associated hospitalizations based on the FluSurv‐NET modeling approach as described in Section 2.6. The solid blue line in Figure 5 shows the median estimate for the detection uncertainty of national COVID‐19‐associated admission rates, whereas the blue band reflects a 95% uncertainty interval corresponding only to the lack of precise knowledge on testing frequency and sensitivity. A comparison of Figures 5 and 1 indicates that not addressing extrapolation uncertainty leads to substantially narrower estimated uncertainty intervals for the assumed national rates. This difference provides evidence that ignoring the impact of extrapolation likely results in potentially substantial underestimation of uncertainty associated with the estimated national rates. The reported NHSN admission rates are also presented in Figure 5. Although the majority of NHSN reported rates fell within the 95% uncertainty interval for the model estimates in Figure 1, this is not the case when extrapolation uncertainty is not addressed (Figure 5) with the majority of NHSN reported rates falling outside the estimated 95% intervals for the detection uncertainty only approach. If one interprets the uncertainty intervals as estimated feasible ranges for the true rates, then a comparison of Figures 1 and 5 indicates greater agreement with NHSN when the modeling approach addresses extrapolation uncertainty.

## 4 Discussion

Data from an active, population‐based sentinel surveillance system, in combination with a Bayesian modeling approach, was used to estimate that from September 2020 through December 2023, 6.3 million (95% UI 5.4 to 7.3 million) COVID‐19‐associated hospitalizations occurred in the United States. This estimate is in close agreement with that reported through the NHSN system, providing evidence that data from this large, geographically diverse sentinel surveillance system can be used to estimate the burden of COVID‐19 hospitalizations in the United States.

Using data from a sentinel surveillance system to estimate national level outcomes critically relies on an assumption that the catchment population is reasonably representative of the US population. The COVID‐NET system was designed to provide wide geographical coverage with a participating site located within each US Health and Human Services region for the majority of the study period. The assumption of representativeness in the COVID‐NET data, however, is not verifiable beyond empirical evidence such as comparisons to other sources, for example, the NHSN data. As a result, interpretation of the extrapolated rates should be conducted with this limitation in mind. In addition, reliance on an unverifiable assumption of representativeness argues, at a minimum, for the need to consider uncertainty due to extrapolating from the catchment to national populations when evaluating sentinel surveillance‐based estimates. The importance of modeling extrapolation uncertainty is illustrated by the substantial reduction in the width of the estimated uncertainty intervals when extrapolation uncertainty is ignored (Figure 5) as opposed to when this important component of total uncertainty is addressed (Figure 1).

Comparison of admission rates estimated using the CAR‐RE model and those reported in the NHSN suggests general agreement both in the magnitude and temporal trend between these surveillance approaches. However, rates reported in NHSN were greater than those estimated hospitalizations using the CAR‐RE model during some time intervals, for example, during the surge in hospitalizations occurring in August and September 2021 and in January 2022. This exceedance of NHSN reported rates over those estimated via the CAR‐RE model likely reflects an unknown combination of two factors. First, it is quite possible that rates in areas outside COVIDS‐NET catchment area exceeded those observed among COVID‐NET site, especially during these periods of surges in hospitalization admissions. Second, case definitions and verification procedures differed between the two surveillance systems. As in COVID‐NET, guidance provided to hospitals reporting COVID‐19 hospitalizations within the NHSN includes a requirement for a laboratory‐confirmed positive test for SARS‐CoV‐2 infection [12]. However, no adjustment is made to NHSN reported counts to address the probability of underdetection because of testing frequency and performance. Because the CAR‐RE modeled estimates are adjusted for estimated underdetection, one would expect the CAR‐RE estimates to be greater than those reported in the NHSN. The results illustrated in Figure 1 indicate that, although this appears to be the case later in the estimation interval, in some earlier periods, the NHSN reported rates exceed those estimated using the CAR‐RE model. One potential explanation for this apparently inconsistent result is uncertainty in the NHSN data due to unknown differences in methods of defining a COVID admission among participating NHSN hospitals. For example, NHSN guidance on the definition of a laboratory‐confirmed test provides no time frame for when that test should have occurred, and cases readmitted shortly after discharge may be counted as two hospitalizations. For COVID‐NET eligibility, however, laboratory confirmation is required either during inpatient stay or within 14 days prior to admission, and COVID‐NET does not include readmissions less than 14 days of a prior COVID‐19‐associated hospitalization as separate case. In addition, potential COVID‐19‐assocated admission within the COVID‐NET system are reviewed by trained surveillance officers to ensure they meet the case definition [1]. Such case review methodology is not universally required for hospitals reporting to NHSN [12]. The largest discrepancies between the NHSN and CAR‐RE rates occurred in time periods associated with rapid increase in hospitalizations; it is plausible, that, during such surge periods, the ability to verify laboratory confirmation of a positive SARS‐CoV‐2 test becomes difficult in the hospital setting, and as a result, presumptive COVID cases may be counted among the NHSN admissions. However, the fact that, in general, the NHSN reported rates fall within the estimated uncertainty bounds produced using the CAR‐RE model, even during surges in hospital admissions, provides additional rationale for the need to address uncertainty in the COVID‐NET‐based estimates resulting from extrapolation from the catchment to national populations.

A previous Bayesian modeling approach has been presented in which COVID‐NET surveillance data were used to estimate national COVID‐associated hospital admission rates [3]. This model, however, was based on an assumption that the observed COVID‐NET rates were independent across months. That is, the modeling did not address the time series nature of the reported monthly COVID‐NET counts. In addition, the earlier model was independently fit within age classes, implying an additional assumption of independence in the observed COVID‐19‐hospitalization rates across age categories. Empirical evidence indicating the presence of temporal dependence, within site clustering, and correlation in the rates across age groups in the COVID‐NET data is presented in Appendix accompanying this manuscript. As a result, it is likely that modeling that does not address these potential sources of correlation can lead underestimation of the uncertainties associated with the estimated rates. This underestimation can be especially problematic when the correlated estimates are summed across states and age classes to produce national estimates. As a result, the CAR‐RE model presented here can be considered as an extension of this earlier work in which dependence is addressed both across time and age class, and within COVID‐NET sites.

Although the CAR‐RE model provides a method to estimate national‐level COVID hospital admission counts and rates, the method is not structured to provide state‐level estimates. This lack of specificity reflects the assumption of a common distribution for state‐level REs and model‐level variances as indicated in Equation (3). Future work on evaluating potential associations between COVID‐NET site‐level admission rates and demographic or other factors in the associated catchment areas may provide insight to guide the development of state‐level admission estimates using the CAR‐RE modeling approach [3].

Another limitation in the current approach is that COVID‐NET testing frequency data were limited to the time period ending in September 2021. This lack of data results in the need to assume testing practices among COVID‐NET hospitals after September 2021 are similar to those observed in the 6‐month period prior to that date. If testing practices within the COVID‐NET hospitals changed meaningfully after September 2021, for example, if universal testing for SARS‐CoV‐2 for hospitalized patients increased, then the approach could lead to overestimation of national rates in these later periods. More recent testing practice data are currently being collected within COVID‐NET hospitals, and the presented estimates will be updated to reflect these data when they become available.

A further limitation in the CAR‐RE extrapolation approach is that potential correlation among the state‐level extrapolated rates due to spatial proximity is not addressed in sampling the posterior predictive estimates of the state‐level admission rates. The primary reason for not incorporating potential spatial dependence is that data to quantify the magnitude of such dependence due to the state‐level spatial structure are not currently available.

The CAR‐RE modeling approach facilitates use of COVID‐NET surveillance data to estimate national COVID‐19‐associated hospitalization rates in which the resulting estimates reflect uncertainty due to extrapolation from catchment to national populations. The CAR‐RE model estimates aligned closely with NHSN national reporting, indicating that this sentinel surveillance system can be used to accurately follow national trends in COVID‐19‐associated hospitalizations. In addition, the importance of such alternative COVID‐19 burden estimation approaches, like the CAR‐RE model, will likely increase given the termination of required mandatory reporting of COVID‐19‐associated hospitalizations to NHSN in April of 2024 [12].

The CAR‐RE model is likely applicable to other diseases/infections currently surveilled using sentinel hospitalization data, for example, RSV‐related hospitalizations [13]. In addition, given the transparent assumptions underlying the use of the CAR‐RE model, that is, that the average rates observed in the catchment population, and the variation of the site‐specific rates about that average, provide representative estimators for corresponding national values, the suggested approach provides a general framework for estimating the uncertainty associated with extrapolation of data observed in sentinel surveillance systems when such systems can reasonably be assumed to be representative of national‐level trends. Such applications can lead to better understanding of the comparative hospital burden of co‐circulating respiratory infections and inform prioritization of public health messaging, interventions, and resources to address these issues.

## Author Contributions

O Devine conceived the modeling approach, developed the model, wrote first draft of the manuscript, and edited revisions to reflect coauthor comments. H Pham provided COVID‐NET data and participated in model development. B Gunnel, HE Reese, and M Steele provided advice on model concept and development. F Havers provided advice on model concept and development and oversaw project progress. All authors provided comments on earlier versions of the manuscript.

## Disclosure

The findings and conclusions in this manuscript are those of the author(s) and do not necessarily represent the official position of the Centers for Disease Control and Prevention (CDC).

## Conflicts of Interest

Meek J declares CDC funding for Connecticut Emerging Infections Program, Ryan PA declares salary support from an Emerging Infections program cooperative agreement with CDC, Reeg L declares funding from a CSTE/CDC federal grant, and Resini B declares receipt of honoraria from Merck for editorial board membership.

### Peer Review

The peer review history for this article is available at https://www.webofscience.com/api/gateway/wos/peer‐review/10.1111/irv.70026.

## Supporting information


**Figure S1** Differences between the observed monthly COVID‐NET site‐specific log admission rates and the average log admission rates across sites by age class.
**Figure S2** Observed association^1^ between monthly COVID‐NET site‐specific log admission rates and the average residual log rates^2^ in temporally adjacent months by age class.
**Figure S3** Observed association^1^ between age class‐specific COVID‐NET log admission rates and the average residual log rates^2^ in adjacent age classes.

## Data Availability

Data are not publicly available. Please contact the corresponding author, Owen Devine (ojd1@cdc.gov), with data‐related questions.
