# Supplementary material for: Extrapolating Sentinel Surveillance Information to Estimate National COVID Hospital Admission Rates: A Bayesian Modeling Approach
Source: Influenza Other Respir Viruses. 2024 Oct 23;18(10):e70026. doi: 10.1111/irv.70026 (PMC11497105; doi:10.1111/irv.70026)
Supplement: Supplementary file 1 — Figure S1 Differences between the observed monthly COVID‐NET site‐specific log admission rates and the average log admission rates across sites by age class. Figure S2 Observed association1 between monthly COVID‐NET site‐specific log admission rates and the average residual log rates2 in temporally adjacent months by age class. Figure S3 Observed association1 between age class‐specific COVID‐NET log admission rates and the average residual log rates2 in adjacent age classes. [file IRV-18-e70026-s001.docx]

**Extrapolating Sentinel Surveillance Information to Estimate National COVID Hospital Admission Rates: A Bayesian Modeling Approach**

**APPENDIX**

**Adjusting COVID-NET hospitalization counts to reflect testing frequency and test sensitivity**

The first step in the modeling process was to adjust the observed site-specific COVD-NET reported COVID-associated hospitalization counts to reflect potential under detection due to non-universal testing for SARS-CoV-2 and the sensitivity of the utilized tests.^1^ Enumeration as a COVID-NET admission requires a confirmed laboratory test for SARS-CoV-2 either during the hospital stay or within 14 days prior to admission. For this analysis, tests used to detect SARS-CoV-2 infection are assumed to be fully specific. That is, the probability of a false positive result is considered negligible. Therefore, to be counted as a COVID-NET eligible admission, an inpatient must be infected with SARS-CoV-2, admitted to a participating COVID-NET hospital, tested for SARS-CoV-2 infection, and have a positive test result. Let $P( +, H, T, T^{+} )$represent the probability of this combination of events where $+$ represents infection with SARS-CoV-2, $H$ being admitted to the hospital, $T$ testing for infection, and $T^{+}$a positive test result.

The goal in adjusting the observed COVID-NET hospitalization counts was to derive an estimator for $P( +, H )$ , that is, the probability of being both admitted to the hospital and being infected with SARS-CoV-2. The rational for the proposed adjustment approach is based on the conditional expansion for $P( +, H, T, T^{+}$ ) in that

$P\left( +, H, T, T^{+} \right)= P\left( +, H )*P( T^{+} \right| T, +, H )*P\left( T \right| +, H )$ . A[1]

Given that one must be tested to have a positive result and under the assumption that the sensitivity of the test is independent of hospitalization status, it follows that

$P\left( T^{+} \right| T, +, H ) = P\left( T^{+} \right| + )$ .

In addition, if the probability of testing and test sensitivity are considered independent, then dividing the conditional probability in equation A[1] by the product

$P\left( T^{+} \right| + )*P\left( T \right|+, H )$ A{2]

yields the desired joint probability of infection and hospital admission. Estimated values for the product of the probabilities given in equation A[2] were used as an adjustment factor for the observed COVID-NET admission counts and will be referred to as the detection probability for COVID-19-associated admissions.

Data collected in a separate sampling process within 11 of the 14 participating COVID-NET sites was used to estimate $P\left( T \right| + , H )$.^2^ These data were based on a random sample of inpatients admitted with a recorded pneumonia, influenza or COVID-19 like condition (PIC) between April 2020 and September 2021. The observed proportion of sampled records in which the patient was reported to have been tested for SARS-CoV-2, either during the hospital stay or within 14 days prior to admission, and the type of test used, was recorded within month and age class (<18, 18-49, 50-64, 65-74, 75-84, 85+ years) for each participating site. By restricting estimation of testing frequency to the hospitalized PIC population, it can be argued that the probability of testing is independent of true SARS-CoV-2 infection status and, therefore, that observed proportion of PIC patients who were tested is an unbiased estimator for $P\left( T \right|+ , H )$ in equation A[2]. Due to small sample sizes within site, month and age class strata, estimates were derived by combining the monthly data into three six-month time intervals, April 2020 through September 2020, October 2020 through March 2021 and April 2021 through September 2021.

Uncertainty concerning $P\left( T \right|+, H )$ within each six-month time interval was modeled by assuming a site and age class-specific Beta prior distribution for this probability. To illustrate, let $s, s=1, .., 11,$reflect site for the COVID-NET locations providing data on testing frequency, $a$ indicate age class, and $m$ month, ranging from April 2020 through September 2021. In addition, let ${pt}_{msa}$ represent the probability of testing for SARS-CoV-2 for each month, age class and site. Note that, within each site and age class, ${pt}_{msa}$ values are identical for months falling within each six-month time interval. Estimates for ${pt}_{msa}$ were defined as the observed testing frequency within each site, age-class and 6-month time interval. The Beta prior assumed for ${pt}_{msa}$, was defined as

${pt}_{msa} \sim Beta( \alpha_{msa} , \beta_{msa} )$ A[3]

with values for $\alpha_{msa}$ and $\beta_{msa}$ selected to reproduce the observed value and the sample variance for stratum-specific testing frequency.^3^ The median value for the observed testing frequencies across COVID-NET sites reporting testing information was used to estimate testing probability for the three sites not reporting these data. For months later than September 2021, ${pt}_{msa}$, and the uncertainty associated with this probability, was assumed to equal that estimated for the last six-month period during which these data were available, April through September 2021.

Evaluation of the data on the type of tests used in the COVID-NET sites indicated that over 97% of all SARS-CoV-2 tests administered between April 2020 and September 2021 were either molecular assay ($MOL)$ or rapid antigen ($RAT).$ Because of the dominance of these two test types in the sample data and because testing with either a molecular assay or rapid antigen is a requirement for inclusion of an admission in COVID-NET, the testing types were limited to molecular assay and rapid antigen only.

An overall test sensitivity, $Se$, within each six-month interval, was estimated as a weighted average of the assumed $MOL$ and $RAT$ test sensitivities where

${Se}_{msa}= {wMOL}_{msa}*{Se}_{MOL} + {wRAT}_{msa}*{Se}_{RAT}$ . A[4]

In equation A[4], ${Se}_{msa}$ reflects the average sensitivity of tests for SAR-CoV-2 infection administered in site $s$, age class $a$, and month $m$, ${wMOL}_{msa}$ is the proportion of tests within site, age class and month that were molecular assay, and

${{wRAT}_{msa} = 1 - wMOL}_{msa}$ .

A Beta prior distribution was assumed for ${wMOL}_{msa}$such that

${wMOL}_{msa} \sim Beta( {\alpha MOL}_{msa} , {\beta MOL}_{msa} )$

with prior values for the parameters ${\alpha MOL}_{msa}$ and ${\beta MOL}_{msa}$selected to reproduce the observed stratum-specific proportion and sample variance of tests that were molecular assay. As with the testing frequency estimates, the median proportion of reported molecular assay tests across sites reporting test type data was used to estimate test type uncertainty for sites that did not provide these data and estimates for months beyond September 2021 were assumed to equal those observed during the last six-month period in which test type data was reported.

Based on available literature,^4-10^ the assumed uncertainty for the sensitivity of the $MOL$ and $RAT$ tests were modeld using uniform distributions such that

${Se}_{MOL} \sim U(0.85, 0.98)$

and

${Se}_{RAT} \sim U(0.65, 0.75$ ) .

Let ${pd}_{msa}$ represent the site, age class and month-specific estimator for the probability, given in equation A[2], that an inpatient with a SARS-CoV-2 infection admitted to a COVID-NET facility is both tested for infection and that the test produces a correct positive result. Using the, assumed independent, estimates given in equations A[3] and A[4], ${pd}_{msa}$ was estimated as

${pd}_{msa} = {pt}_{msa}* {Se}_{msa}$ . A[5]

**Estimation of temporal trends in monthly COVID-NET hospitalization rates**

The COVID-NET data used in this analysis was comprised of monthly counts of eligible admissions to participating hospital sites reported from April 2020 through December 2023. In addition, estimates of the appropriate catchment population size at risk for admission to each hospital were reported for each site.^11^ Admission counts and catchment populations sizes were reported within each of six age classes, <18, 18-49, 50-64, 65-74, 75-84, and 85+ years, from April 2020 through May 2022 for 14 sites and from 13 sites from June 2022 through December 2023. The reduction in the number of participating sites is due to information from one site no longer being available after May 2022. However, COVID-associated hospitalizations counts and catchment populations for the site in question were included as model inputs from April 2020 through May 2022.

As a first step in the modeling process, the monthly site and age-class specific admission counts were adjusted, using the parameter ${pd}_{msa}$ given in equation A[5], to reflect the number of inpatients who would have a COVID diagnoses if all persons with a PIC were tested and the test had perfect sensitivity. Let $c_{msa}^{obs}$ be the reported number of hospital admissions meeting the COVID-NET testing requirement for month $m$, site $s$, and age class $a$. In addition, let $c_{msa}$ be the unobserved number of COVID-admissions if all inpatients with a PIC were tested using a fully sensitive test. The observed admission count is modeled as the outcome of a binomial trial such that

$c_{msa}^{obs} \sim Bin( {pd}_{msa} , c_{msa} )$ . A[6]

implying that the observed COVID admission counts follow a binomial distribution with probability of success equal to the detection probability given in equation A[5] and number of trials corresponding to the unobserved admission count given complete and perfect testing. Note that $c_{mas}$ is considered missing data that will be estimated using the Bayesian model outlined below.

In the next hierarchy of the model, $c_{msa}$ is assumed to be a sample from the Poisson distribution

$c_{msa}\sim Pois\left( \mu_{msa},{pop}_{msa} \right)$

where ${pop}_{msa}$ is the catchment population size for month $m$, site $s$, and age class $a$ and $\mu_{msa}$ is the corresponding rate of COVID admissions. The natural log, denoted here as $log$(), of $\mu_{msa}$ is modeled as a sample from a Normal distribution under the assumption

$\log\left( \mu_{msa} \right) \sim N( {l\mu}_{ma}+ \varepsilon_{s} , \sigma_{ma}^{2}$) A[7]

where ${l\mu}_{ma}$ is the average log admission rate for month $m$ within age class $a$, $\varepsilon_{s}$ is a site-level random effect, and $\sigma_{ma}^{2}$ is the month and age class-specific model-level variance. The collection of site-level random effects in equation A[7] are assumed to be samples from the Normal distribution

$\varepsilon_{s} \sim N( 0 , \delta^{2} )$ A[8]

with a twofold rationale for including these effects in the model. First, it is likely that some level of clustering among monthly COVID admission rates will occur within sites and that the correlation among the log rates within sites will differ from that between sites. In addition, there are likely site-level factors that influence admission rates that are not accounted for in the available COVID-NET data. To illustrate this second point, Figure A1 shows the differences between the observed log monthly admission rates for each site and the corresponding across site average log rate within each of the six age groups. Notice that the differences from the average log rate are not randomly distributed about zero for all sites. In addition, these site-level differences in the distributions, for example in GA, OH, OR and TN, appear consistent across age groups indicting the likely impact of unmeasured site-level factors.

The vector containing the collection of monthly age class-specific log rates was assumed to be a sample from the multivariate Normal distribution

$\vec{l\mu_{ma}} \sim MVN ( \vec{{l\mu}_{a}} , \Sigma_{a} )$. A[9]

In equation A[9], $\vec{{l\mu}_{ma}}$ denotes a vector of length six, i.e., the number of age classes, with each element corresponding to the age class-specific monthly log admission rate, $\vec{l\mu_{a}}$ is a vector with elements corresponding to the age class-specific average log rate across all months, and $\Sigma_{a}$ is a covariance matrix reflecting the temporal dependence among the elements of $\vec{{l\mu}_{ma}}$. For these analyses, $\Sigma_{a}$ is assumed to correspond to the covariance matrix associated with a one-dimensional proper conditional autoregressive (CAR) model.^12,13^ To illustrate the rationale for this temporal dependence assumption, let ${l\mu}_{ma}- {lu}_{a}$, that is the difference in the age-specific monthly log rate and the average log age-specific rate across all months, be defined as the residual log rate for month $m$. Under the one-dimensional CAR assumption, the conditional distribution of ${l\mu}_{ma}$ given ${l\mu}_{-ma}$, where ${l\mu}_{-ma}$ represents all other monthly log rates excluding ${l\mu}_{ma}$, depends only on the residual log rate in adjacent months. That is, for month in $m=1,..,M$, the conditional distribution of ${l\mu}_{ma}$ given ${l\mu}_{-ma}$ is given by^14^

for $m$= 1,

${l\mu}_{-m,a}, \lambda_{a}, \alpha_{a}^{2}$ ~ $l\mu_{1,a} | l\mu_{-m,a}, \lambda_{a}, \alpha_{a}^{2} \sim N( l\mu_{a} + \lambda_{a} \left( l\mu_{2,a} - {l\mu}_{a} \right) , \alpha_{a}^{2} )$ ,

for $m$ = $M$,

$l\mu_{M,a} | l\mu_{-m,a}, \lambda_{a}, \alpha_{a}^{2} \sim N( l\mu_{a} + \lambda_{a} \left( l\mu_{M-1,a} - l\mu_{a} \right) , \alpha_{a}^{2} )$ , A[10]

and for $m$ = 2, … , $M-1$,

$l\mu_{m,a} | l\mu_{-m,a}, \lambda_{a}, \alpha_{a}^{2} \sim N\left( {l\mu}_{a} + \lambda_{a}\frac{\left( \left( l\mu_{m-1,a}-{l\mu}_{a} \right) + \left( {l\mu}_{m+1, a}-{l\mu}_{a} \right) \right)}{2} , \frac{\alpha_{a}^{2}}{2} \right).$

In equation A[10], the parameter $\alpha_{a}^{2}$ is the conditional variance of ${l\mu}_{ma}$ given all other monthly log rates within age class $a$ while $\lambda_{a}$ reflects the strength of the linear association between $l\mu_{ma}$ and the average of the residual log rates in the adjacent months. Figure A2 shows the results of exploratory analyses conducted to evaluate the plausibility of the conditional dependency assumptions presented in equation A[10]. The figure shows graphs of the observed COVID-NET site level log rates for each month plotted against the average of the residual log rate values observed in the adjacent months, with appropriate adjustment for $m$= 1 and $m=M$. In addition, a linear regression estimate fit to these data is shown to assess the implied linear association under a proper CAR model. This collection of graphs indicates a strong linear association between the log rates and the temporally adjacent residual log rate values for all age classes. An additional advantage of assuming the proper form for the CAR model is that it leads to a closed form estimator for the covariance matrix $\Sigma_{a}.$^12^ This closed form results in computational flexibility in terms of embedding temporal dependency assumptions among the elements of $\vec{l\mu_{ma}}$ within the hierarchical model for $c_{msa}^{obs.}$.

The final level of the proposed model reflects assumptions on the vector of average age class-specific log admission rates across all months, $\vec{{l\mu}_{a}}$ in equation A[9]. To account for potential dependence among the age class-level average log rates, it was assumed that

$\vec{l\mu_{a}} \sim MVN( l\mu*\vec{1} , \Theta$ ) A[11]

where $l\mu$ is the single valued parameter reflecting the average log rate across all months and age classes, $\vec{1}$ is a vector of ones of length six and $\Theta$ is a covariance matrix. To address potential dependence among the age class-specific average log rates across age groups, $\Theta$ was assumed to correspond to the covariance matrix implied under a proper CAR assumption. Figure A3 summarizes exploratory analyses in which average age class-specific mean observed log rates are plotted against the average residual log rates, now defined in terms of age classes, of the adjacent age groups with appropriate boundary modifications for ages <18 and 85+. As with the exploratory assessment of temporal dependence in the monthly log rates, the apparent linear association shown in the graph provides a rationale for the assumed CAR structure among the age class-specific average log rates.

To summarize, the hierarchical model used to estimate the parameters governing temporal trends in the unobserved true number of COVID-NET hospital admissions infected with SARS-CoV-2 and the variation in site-level log rates about that average is given by

$c_{msa}^{obs} \sim Bin( {pd}_{msa} , c_{msa} )$ ,

$c_{msa}\sim Pois\left( \mu_{msa},{Pop}_{msa} \right)$ ,

$\ln\left( \mu_{msa} \right) \sim N( l\mu_{ma}+ \varepsilon_{s} , \sigma_{ma}^{2}$) ,

where

$\varepsilon_{s} \sim N( 0 , \delta^{2} )$ .

Define the vector

$\vec{{l\mu}_{ma}} =( {l\mu}_{1a}, {l\mu}_{2a}, . . . , {l\mu}_{Ma} )$,

and assume it is a sample from a multivariate Normal distribution such that

$\vec{{l\mu}_{ma}} \sim MVN ( \vec{{l\mu}_{a}} , \Sigma_{a} )$,

where

$\vec{{l\mu}_{a}} \sim MVN( log(\mu)*\vec{1} , \Theta$ ) . A[12]

In addition, define

${pd}_{msa} = {pt}_{msa}* {Se}_{msa}$

where

${pt}_{msa \sim}Beta( \alpha_{msa} , \beta_{msa} )$ ,

${Se}_{msa}= {wMOL}_{msa}*{Se}_{MOL} + {wRAT}_{msa}*{Se}_{RAT}$ ,

${wMOL}_{msa} \sim Beta( {\alpha MOL}_{msa} , {\beta MOL}_{msa} )$ ,

${wRAT}_{msa} \sim1 - {WMOL}_{msa}$ ,

with

${Se}_{MOL} \sim U(0.85, 0.98)$

and

${Se}_{RAT} \sim U(0.65, 0.75$) .

To complete specification of the model, the prior distribution for the standard error of the site level random effects was assumed to be uniform^15^ such that

$\delta\sim U(0,100)$.

Prior assumptions for the parameters of the CAR model described in equation A[10] were

$$\lambda_{a} \sim U(-1, 1 )$$

and

$\alpha_{a} \sim U(0,10)$

with identical priors used for the corresponding dependence and variance parameters in the CAR model for potential correlation across age class average log rates. The parameter $\mu$ in equation A[12] corresponds to the mean hospitalization rate across all months, sites and age classes and was assumed to have a prior distribution given by

$\mu\sim U\left[ 0.0001, 0.2 \right] .$ A[13]

Note that, given the noninformative prior in equation A[13], the overall average hospitalization rate is assumed a priori to fall anywhere between 1 and 2000 per 10,000 population.

Due to the use of an assumed proper CAR model for temporal and across age-class dependence and incorporation of site-specific random effects, the proposed model will be referred to as the CAR-RE model.

The data were processed using R (version 4.2.2) software with the Bayesian model fit using JAGS (Version 4.3.1) under the R2Jags interface ([CRAN - Package R2jags (r-project.org)](https://cran.r-project.org/web/packages/R2jags/index.html). One sampling chain was run for 45,000 iterations with the initial 5000 samples excluded as a burn in phase. The sampling chain of length 40,000 was then thinned by selecting every second sample leading to a final sample of 20,000 posterior samples. Due to the number of parameters estimated, convergence assessment based on comparison of multiple chains was challenging. However, additional runs of the model using multiple chains were conducted with a subset of output estimands retained. These quality assurance runs indicated that convergence appeared evident well before the burn in of 5000 samples that was used in the estimation process.

**Extrapolation based on CAR-RE posterior estimates**

Extrapolation of the modeled COVID-NET site rates to the U.S. national level was based on two key assumptions: 1. The estimated collection of monthly age class-specific rates among COVID-NET sites provides an unbiased estimate for the corresponding U.S. national age-specific rates and 2. The distribution of COVID-NET age and site-specific rates about that monthly average reflects the expected distribution of state and age class-specific rates about the national age-specific average.

Let the superscript $(i)$ indicate the $i$th posterior sample produced using the CAR-RE model where $i$= 1,.., 20,000. The first step in process used to estimate national admission rates is to sample the $i$th posterior value for the vector of monthly age class-specific log admission rates, $\vec{{l\mu}_{ma}^{(i)}} ,$and the associated $i$th posterior estimate for the variance of the site-level random effects, $\delta^{2(i)}$. Let the bolded capitalized subscript $\boldsymbol{S}$ designate a given state as opposed to the small un-bolded $s$ which, so far, has been used to designate COVID-NET sites. An estimated value for a random effect associated with each state was sampled as

$\varepsilon_{\boldsymbol{S}}^{(i)} \sim N( 0 , \delta^{2\left( i \right)} )$ A[12]

for each of 50 states and the District of Columbia. Note that the elements of the vector $\vec{{l\mu}_{ma}^{(i)}}$ correspond to posterior estimates of the age class specific monthly log admission rates from month 1 through month $M$ where

$$\vec{{l\mu}_{ma}^{(i)}} = \left( {l\mu}_{1a}^{\left( i \right)} ,{l\mu}_{2a}^{\left( i \right)},\ldots, {l\mu}_{Ma}^{\left( i \right)} \right) .$$

For each monthly log rate, that is, for each element in the sampled vector, the estimated state-level log admission rate for age class $a$ was then sampled from the distribution

$\log\left( \mu_{m\boldsymbol{S}a}^{(i)} \right) \sim N( {l\mu}_{ma}^{\left( i \right)} + \varepsilon_{\boldsymbol{S}}^{\left( i \right)} , \sigma_{ma}^{2\left( i \right)} )$ A[13]

where $\sigma_{ma(i)}^{2}$ is the $i^{th}$ posterior sample for the month and age class-specific model-level variance parameter. Under an assumption that the variance of the state-level random effects and the model-level variance are independent, the sampling assumption in equation A[13] can be re-written as

$\log\left( \mu_{m\boldsymbol{S}a}^{(i)} \right) \sim N( {l\mu}_{ma}^{\left( i \right)} , \delta^{2\left( i \right)}+\sigma_{ma}^{2\left( i \right)} )$ . A[14]

The sampling distribution in equation A[14] illustrates the two primary assumptions underlying estimation of national admission rates using the counts observed in the COVID-NET data. First, that the average monthly age-specific log rates estimated in the COVID-NET data provide unbiased estimators for the corresponding national age-specific rates and, second, that the variance among state-level rates about the national monthly average can be estimated by the corresponding distribution of site-level rates about the COVID-NET monthly average. That is, that variance of the monthly state-level age-specific log admission rates about the national average log rates can be estimated as $\delta^{2\left( i \right)}+\sigma_{ma}^{2\left( i \right)}$.

Based on the estimated values for the state and age-specific log admission rates derived using equation A[13], a collection of 20,000 estimates for the extrapolated admission counts for $\boldsymbol{S}$ = 1, …, 51, 50 states and the District of Columbia, was then generated under the Poisson assumption

$c_{m\boldsymbol{S}a}^{(i)} \sim Pois\left( \mu_{m\boldsymbol{S}a}^{(i)},{Pop}_{\boldsymbol{S}a} \right)$ . A[15]

In equation A[15], $c_{m\boldsymbol{S}a}^{(i)}$ is the $i$th sample for the estimated COVID admission count for month $m$, age class $a$, in state $\boldsymbol{S}$, $\mu_{m\boldsymbol{S}a}^{(i)}$ is defined in equation A[14] and ${Pop}_{\boldsymbol{S}a}$ is the age class specific state population size.^16^ The estimates of age class and state-level hospitalization counts were then summed across states resulting in 20,000 posterior predictive estimates for national hospitalization counts within each month and age class. These age-class specific hospitalization count estimates were also summed across age groups yielding a collection of total national hospitalization count estimates for each month. The estimates were then divided by the appropriate U.S. age-specific population size to derive national estimated hospitalization rates for each month and age class. The resulting distributions of estimates, both national total and national within age class, tended to be skewed to the right. As a result, the age class and overall month-specific hospitalization count and rate estimates were summarized using the median of the 20,000 estimates and a 95% uncertainty interval based on the highest posterior density approach.^17^

REFERENCES

1. Reed C, Chaves S, Kirley P, et al. Estimating influenza disease burden from population-based surveillance data in the United States. *PLoS ONE*. 2015;10(3). <https://doi.org/10.1371/jounrnal.pone.0118369>
2. O’Halloran A, Whitaker M, Patel K, et al.  [Developing a sampling methodology for timely reporting of population-based COVID-19-associated hospitalization surveillance in the United States, COVID-NET 2020–2021](https://onlinelibrary.wiley.com/doi/10.1111/irv.13089). Influenza Other Respi Viruses. 2023;Jan 10; 1-8. <https://doi.org/10.1111/irv.13089>
3. Forbes, C., M. Evans, N. Hastings, and B. Peacock. Statistical Distributions. Fourth Edition. 2011. John Wiley and Sons, Hoboken, NJ.
4. Arshadi M, Farddsanei F, Deihim B, et al., Diagnostic accuracy of rapid antigen tests for COVID-19 Detection: A systematic review with meta-analysis. *Front. Med*. 2022; 9**.** <https://doi.org/10.3389/fmed.2022.870738>
5. Brummer L, Katzenschlager S, McGrath S, et al., Accuracy of rapid point-of-care antigen-based diagnostics for SARS-CoV-2: An updated systematic review and meta-analysis with meta-regression analyzing influencing factors. *PLoS Med*. 2022;19(5). <https://doi.org/10.1371/journal.pmed.1004011>
6. Hohl C, Hau J, Vaillancourt S, et al., Sensitivity and diagnostic yield of the first SARS-CoV-2 nucleic acid amplification test performed for patients presenting to the hospital. *JAMA Network Open*. 2022;5(10). <https://doi.org/10.1001/jamanetworkopen.2202.36288.>
7. Khalid M, Selvam K, Jeffry A, et al., Performance of rapid antigen tests for COVID-19 diagnosis: A systematic review and meta-analysis. *Diagnostics*. 2022;12(110). <https://doi.org/10.3390/diagnostics12010110>
8. Marando M, Tamburello A, Gianella P, et al., Diagnostic sensitivity of RT-PCR assays in nasopharyngeal specimens for detection of SARS-CoV-2 infection: A systematic review and meta-analysis. *Caspian J Inten Med*. 2022;13(Suppl 3):139-147. <https://doi.org/10.22088/cjim.13.0.139>
9. Veroniki A, Tricco A, Watt J, et al., Rapid antigen-based and rapid molecular tests for detection of SARS-CoV-2: A rapid review with network meta-analysis of diagnostic test accuracy studies. *BMC Med*. 2023;21(110). <https://doi.org/10.1186/s12916-023-02810-0>
10. Xie J, He Y, Zheng Y, et al., Diagnostic accuracy of rapid antigen test for SARS-CoV-2: A systematic review and meta-analysis of 166,943 suspected COVID-19 patients. *Microbio Res.* 2022;265. <https://doi.org/10.1016/j.micres.2022.127185>
11. CDC. COVID-NET Overview and Methods.<https://www.cdc.gov/coronavirus/2019-ncov/covid-data/covid-net/purpose-methods.html>
12. Lee D. A comparison of conditional autoregressive models used in Bayesian Disease mopping. *Spat Spatio-temporal Epidemiol.* 2001;2:79-89. <https://doi.org/10.1016/j.sste.2011.03.001>
13. Gelfand A, Vounatsou P. Proper multivariate conditional auto regressive models for spatial data analysis*. Biostatistics*. 20023;4(1). <https://doi.org/10.1093/biostatistics/4.1.11>
14. Shaddick G, Wakefield J. Modelling daily multivariate pollutant data at multiple sites. *Appl Statist*. 2002;**51**(3);351-372. <https://doi.org/10.1111/1467-9876.00273>
15. Gelman A. Prior distributions for variance parameters in hierarchical models. *Bayes Anal.* 2006;1(3):515-533. <https://doi.org/10.1214/06-BA117A>
16. CDC Wonder: bridged-race population estimates. Centers for Disease Control and Prevention. <https://wonder.cdc.gov/bridged-race-population.html>
17. Kruschke, J.. Doing Bayesian data analysis: a tutorial with R and BUGS. 2001. Elsevier, Amsterdam.

Figure A1 Differences between the observed monthly COVID-NET site-specific log admission rates and the average log admission rates across

sites by age class


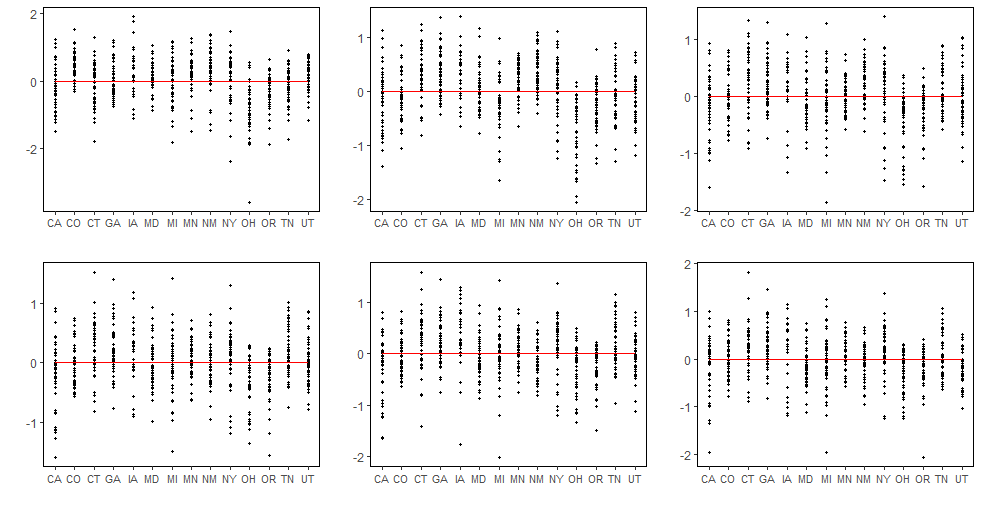


COVID-NET Site

Log(Rate) – Average Log(Rate)

85+ Years

75-84 Years

65-74 Years

59-64 Years

18-49 Years

< 18 Years

Figure A2 Observed association^1^ between monthly COVID-NET site-specific log admission rates and the average residual log rates^2^ in

temporally adjacent months by age class


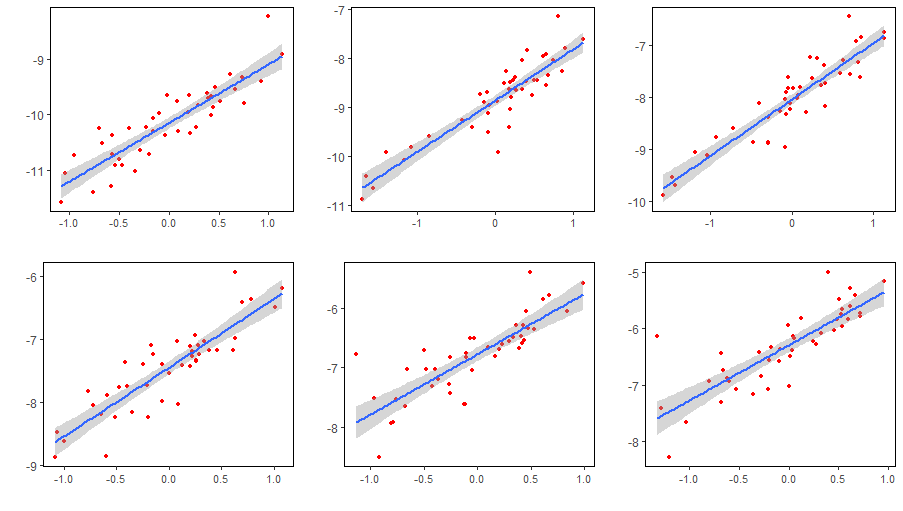


Log(Rate)

Average Residual Log Rate

50-64 Years

18-49 Years

85+ Years

75-84 Years

65-74 Years

< 18 Years

^1^ Line and shaded area correspond to linear regression estimates and 95% confidence intervals.

^2^ The residual log rate is defined as the difference in the observed log rate in each month and the average log rate across all months in the estimation interval.

Figure A3 Observed association^1^ between age class-specific COVID-NET log admission rates and the


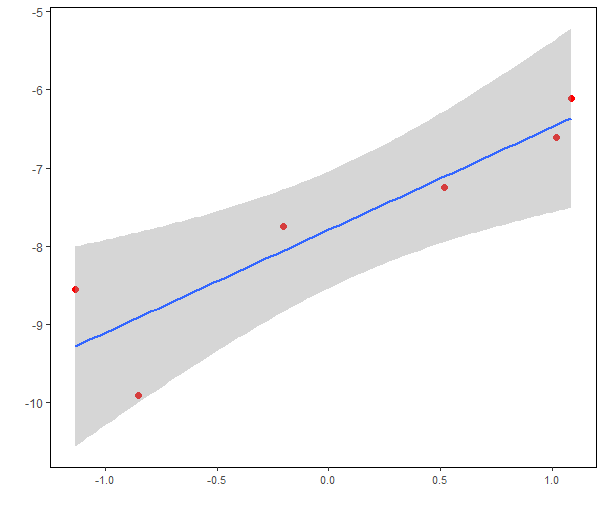
 average residual log rates^2^ in adjacent age classes

Average Residual Log Rate

Log(Rate)

^1^ Line and shaded areas correspond to linear regression estimates and 95% confidence intervals.

^2^ The residual log(rate) is defined as the difference in the observed log(rate) in a given age class and the average log rate across

all age classes.
